# Editorial: Pharmacokinetics, pharmacodynamics (PK/PD) of antibiotics: A reality check

**DOI:** 10.3389/fphar.2023.1150472

**Published:** 2023-02-15

**Authors:** Markus Zeitlinger, Glenn Tillotson, Roger Echols

**Affiliations:** ^1^ Department of Clinical Pharmacology, Medical University of Vienna, Vienna, Austria; ^2^ Infectious Disease Drug Development Consulting, LLC, Easton, CT, United States; ^3^ GST Micro, Henrico, VA, United States

**Keywords:** PK/PD, antibiotic, drug develoment process, betalactam, gram negative

Pharmacokinetic/Pharmacodynamic (PK/PD) principles have become a backbone of antibiotic drug development and dose selection for novel as well established antimicrobials (Palmer et al.). The ultimate aim obviously is to optimize treatment of patients suffering from an infection, but other goals like accelerating access to novel antibiotics and reduction of development costs as well as prevention of development of bacterial resistance and setting of appropriate breakpoints are of high relevance, too.

As seen for every novel approach it took some time to establish applicable PK/PD paradigms, but in parallel the complexity of the models (both *in vitro* and *in vivo*) and the understanding of the processes has increased dramatically. In the present Research Topic prominent experts in the field of PK/PD set out to challenge some of these paradigms, either by a reality check or by improvements of the currently used models.

Since preclinical PK/PD thresholds are a cornerstone of PK/PD studies that are used to determine the best dosing regimen in patients, two manuscripts dealt with PK/PD targets of *β*-Lactam antibiotics. While for meropenem 40% T > MIC is associated with optimal killing of pathogens, Nussbaumer et al. for the first time could demonstrate that the distribution of the %T > MIC periods throughout the day did impact the bacterial eradication. In line with this Berry and Kuti identified the need for targets that are specific to each antibiotic and pathogen, in particular during dosage regimen development and susceptibility breakpoint assessment. Thereby both studies demonstrated that some common paradigms of PD thresholds might be over-simplified.

Although the importance of the bacterial inoculum is well known *in vitro*, in another oversimplification most animal PD models use a standardized inoculum, and few *in vivo* studies have investigated the impact of inoculum size on survival or antibiotic efficacy. The study by Chauzy et al., therefore, set out to investigate the influence of inoculum size on polymyxin B against *A. baumannii* confirming an inoculum effect.

Even if appropriate PK/PD parameters are identified given all the methodological challenges mentioned above, achieving the desired exposure in individual patients remains challenging. Thus, another study on Polymyxin B addressed the problem of correctly dosing this potentially nephrotoxic drug in obese patients, Wang et al. identified an adjusted body weight (ABW)-based regimen that has a high likelihood of achieving the targeted exposure. Alternative to using body weight adapted dosing regimens, TDM (therapeutic drug monitoring) approaches can be used to individualize dosing of antibiotics. Yet, in clinical practice often only sparse PK is available and results may not be timely to impact patient care. Thus, a clinical approach was employed by Cojutti et al. to identify the best target for meropenem when given as continuous infusion for critically ill patients with Gram-negative infections. By classification and regression tree (CART) analysis the authors found that concentration (Css)-to-minimum inhibitory concentration (MIC) (Css/MIC) ratio correlated with favorable clinical outcome.

Last but not least the information gathered by PK/PD scientists has to reach the clinical practice. Often the revision of bacterial breakpoints fail to result in clinical benefit for the patients due to logistic reasons, as demonstrated by Redell and Tillotson for carbapenems and Enterobacterales. A detailed review by Landersdorfer and Nation on MIC-based PK-PD metrics, from historical perspectives to the most modern translational models, summarized both pitfalls but also opportunities in linking antimicrobial susceptibility testing, target site exposure and bacterial response. Major shortcomings of PK/PD were identified by the authors that emphasize the need to critically appraise this field of research. Yet, we must not forget the tremendous achievements that the PK/PD field has brought to a range of patients types suffering from often life threatening infections. Importantly our understanding of PK/PD continues to develop and mathematical modelers will further refine the relationship between host, pathogen and antibiotics in complex models, with a strong focus to individualize treatment in the sense of personalized medicine.

